# Successful treatment of super-refractory focal status epilepticus: Surgery, vagus nerve stimulation, and botox for epilepsia partialis continua

**DOI:** 10.1016/j.ebr.2025.100775

**Published:** 2025-05-02

**Authors:** N. Zalmay, G. Nune, C.N. Heck, K.T. Dao, B.T. Ly, J. Ipe, C.Y. Liu, H.P. Kunhi Veedu

**Affiliations:** a Kern Medical Center, United States; b University of Southern California, United States

**Keywords:** Super-refractory status epilepticus, Surgical resection, Vagus nerve stimulation, Botulinum toxin therapy, Traumatic brain injury, Case report

## Abstract

Super-refractory status epilepticus (SRSE) is a life-threatening condition characterized by persistent seizures lasting beyond seven days despite anesthetic therapy. Managing SRSE is challenging, particularly in cases resistant to conventional treatments. This case report presents a 41-year-old female with prolonged SRSE secondary to focal epilepsy, successfully treated with surgical resection, vagus nerve stimulation (VNS), and botulinum toxin (BoNT) therapy. The patient initially presented with left focal motor status epilepticus and a right frontal intracerebral hematoma. Despite an emergency craniotomy, her seizures persisted, leading to prolonged SRSE. After multiple failed sedation weaning attempts, a stepwise intervention approach was implemented consisting of an Electrocorticography (ECoG)-guided surgical resection of the residual epileptic focus, VNS implantation for long-term seizure modulation, BoNT therapy for residual epilepsia partialis continua (EPC). The combined approach of surgical resection and VNS implantation successfully terminated the status epilepticus. Residual Epilepsia Partialis Continua (EPC) was effectively managed with botulinum toxin therapy (BoNT). This case underscores the potential efficacy of combining surgical resection and VNS implantation in treating SRSE and highlights the beneficial effects of BoNT in managing residual EPC, offering a potential treatment pathway for similar refractory cases.

## Highlights

• Multimodal approach successfully treated super-refractory focal status epilepticus.
• Surgical resection and VNS implantation effectively terminated status epilepticus.
• BoNT therapy reduced residual epilepsia partialis continua symptoms and spasticity.

## 1 Introduction

Super-refractory status epilepticus (SRSE) is a critical and life-threatening neurological condition characterized by prolonged seizures that are resistant to standard treatment protocols. According to the International League Against Epilepsy (ILAE) Task Force, SRSE is defined as status epilepticus lasting beyond seven days with ongoing use of anesthetics or induction of coma [1]. The most common causes of SRSE include brain trauma, infections, stroke, alcohol and other drug-related causes, as well as immunologically mediated disorders. The management of SRSE is particularly challenging and typically involves a staged approach. Traditional therapies include medical management with anti-seizure medications, continuous intravenous anesthetic (civ) agents, and emerging therapies such as the ketogenic diet and treatment with immunosuppressive agents depending on the underlying etiology [2].

Treatment strategies generally depend on the stage of SRSE. SRSE evolves through a progression of stages. Initially, a patient may present with focal or generalized seizures, which can progress to status epilepticus (stage 1 or early status epilepticus) if not controlled. If seizures continue despite initial interventions with benzodiazepines, the condition transitions to established status epilepticus (stage 2) which may be treated with intravenous anti-epileptic drugs. When seizures persist without a response to treatment for up to two hours, the condition transitions to refractory status epilepticus (RSE) (stage 3), requiring intensive management, including anesthesia to achieve EEG burst suppression. Lastly, when super-refractory status epilepticus continues despite treatment with anesthetics for more than 24 h, the condition evolves into super-refractory status epilepticus (SRSE)(stage 4), where more specialized and aggressive interventions are required, which depend on the underlying causes and response to previous treatments [3].

In rare cases of focal prolonged SRSE, a combined approach using emergent focal resective epilepsy surgery and neurostimulation therapies such as Vagus Nerve Stimulation (VNS) or Responsive Neurostimulation (RNS) may be warranted [4]. However, literature on effectively managing prolonged SRSE in the context of traumatic brain injury (TBI) with dual interventions of surgical resection and neurostimulation therapy remains scarce. Moreover, the development and management of residual Epilepsia Partialis Continua (EPC) in rare cases following these interventions is a topic that warrants further study.

A thorough literature search revealed no case reports involving a combined approach of treating prolonged SRSE with surgical resection and subsequent VNS implantation. This case report aims to delineate a rare instance of prolonged SRSE in a patient following traumatic brain injury, treated with decompressive hematoma evacuation, precise resection of the residual cavity guided by electrocorticography (EcoG), and subsequent VNS implantation. The residual EPC was managed with Botulinum toxin (BoNT) therapy, confirmed by EEG. This case demonstrates the efficacy of combined VNS and surgical resection for SRSE and EPC management with BoNT.

Through this case report, we aim to contribute to the existing literature and provide valuable insights into the collective efficacy of using a multimodal approach (VNS implantation and surgical resection) in the treatment of Prolonged SRSE, in addition to demonstrating the possibility of managing EPC using BoNT.

## 2 Case history

A 41-year-old right-handed female presented to the Emergency Department (ED) in a coma following a fall. Her medical history indicated general good health until the age of 34, when she experienced her initial focal motor seizure of unknown etiology, confirmed by negative MRI results and multiple routine EEG examinations. She was placed on Levetiracetam for preventative measures. Over the next seven years, the patient experienced two seizures. At the age of 41, she developed deep vein thrombosis of unknown etiology and was started on Apixaban as oral anticoagulation therapy.

**Fig. 1:**
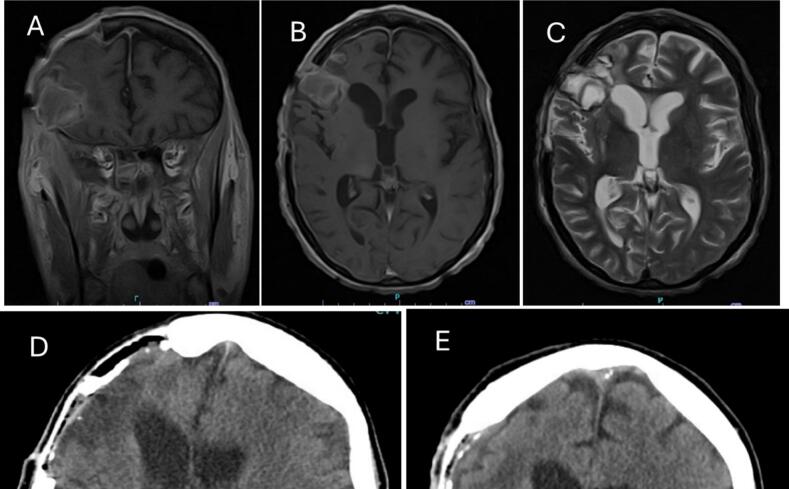
(A) Pre-op Coronal T1 (B) Pre-op Axial T1 (C) Pre-op Axial T2 (D) Post-op Axial CTH (E) Post-op Coronal CTH.

In April 2021, after a fall, the patient was brought to a community hospital where she was found to be in refractory focal status epilepticus characterized by impaired awareness with frequent left hemi-body focal clonic seizures. MRI revealed a traumatic acute right subdural hematoma with cerebral edema and midline shift, severe hemorrhagic contusion of the right frontal lobe, and subarachnoid hemorrhage (Fig. 1a, 1b, 1c). Review of these initial MRI sequences showed no evidence of acute or chronic abnormalities, such as ischemic changes or lesions, within the basal ganglia. An emergency craniotomy was performed for evacuation of the subdural hematoma and removal of the bone flap to control cerebral edema. The patient continued to be in focal status epilepticus. An ICU EEG showed continuous right hemispheric status epilepticus (Fig. 3a). She was treated with IV anesthetics to break the focal status epileptic seizures. After a two-week hospital stay, the focal status epilepticus resolved, and she was discharged to an acute rehabilitation facility on polytherapy including Levetiracetam, phenytoin, and Lacosamide. Four days later, she was readmitted due to repetitive left focal clonic seizures with impaired awareness. An EEG confirmed the patient was in focal status epilepticus. Initial treatment attempts with medical management were unsuccessful, leading to her transfer to the neuro-intensive care unit where she was intubated and sedated with propofol and midazolam to achieve burst suppression on continuous video EEG. Despite multiple attempts to taper off the IV anesthetics, she continued to be in prolonged super-refractory focal status (Fig. 3b). She was transferred to the Neuro-ICU at the University of Southern California. ICU EEG findings revealed the patient is in super-refractory non-convulsive focal status epilepticus and interictal repetitive polyspike wave discharges from the right frontocentral region, consistent with persistent super-refractory non-convulsive right hemispheric focal status epilepticus.

**Fig. 2:**
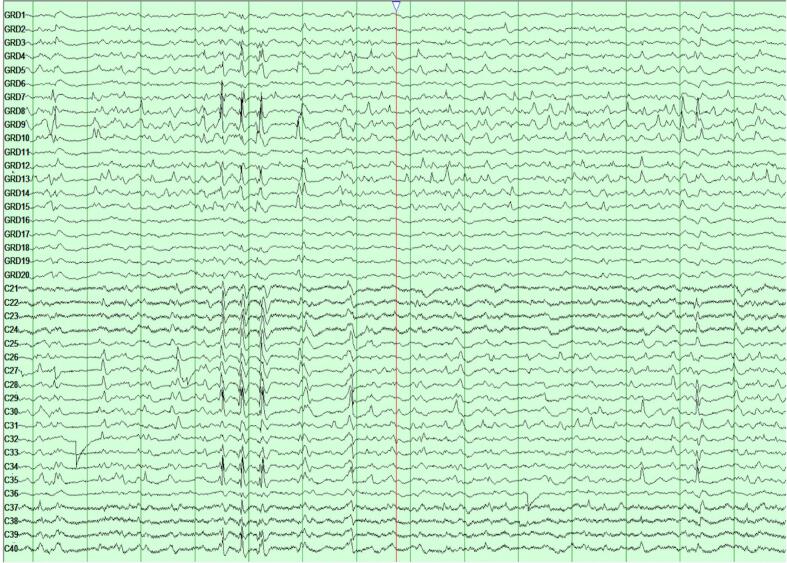
*Pre-resection Electrocorticography (ECoG).* Electrocorticography (ECoG), prior to resection at USC. Two Integra 4x5 grids with 10 mm inter-contact spacing were placed in the right frontoparietal parasagittal and right anterior-middle-posterior temporal regions within the resection cavity. Multifocal spikes are present, some are polyphasic and very broad affecting large parts of the recording grids. The most consistent foci with repetitive spiking were Grid 1 (inferior frontal resection cavity): channels 8, 9, 7, 13, and 14; and to a lesser extent, channels 4 and 5. Grid 2 (superior frontal resection cavity): channels 6, 7, 8, and 11.

**Fig. 3:**
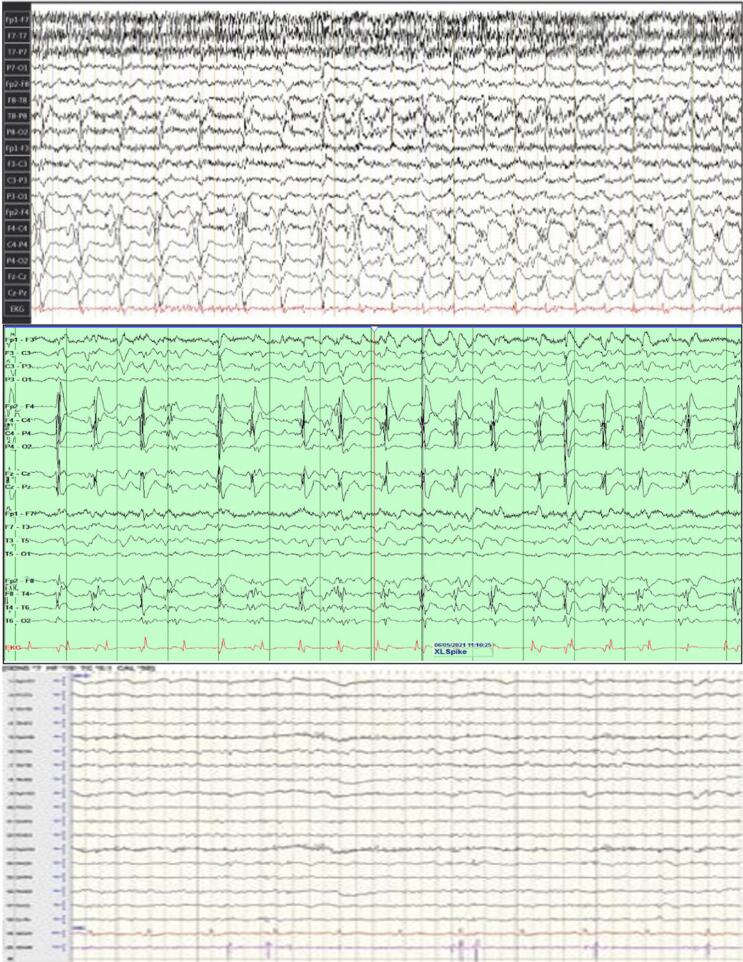
Scalp Electrocochleography (EEG) Recordings. Representative EEG Epochs shown. (A) EEG prior to transfer to USC, showing highly abnormal pattern prominent muscle artifact overlaying suspected epileptiform activity/severe background slowing. (B) EEG during weaning off sedation, demonstrating frequent generalized epileptiform discharges. (C) EEG after (BoNT) treatment, showing significant reduction in epileptiform discharges compared to (B). EEG Display Parameters (for A & B): Sensitivity 7 µV/mm, Low-Frequency Filter (LFF) 1.0 Hz, High-Frequency Filter (HFF) 70 Hz, Notch Filter 60 Hz ON, System: Cadwell Essentia (A) and Xltek EEG Machines (B). Original Sampling Rate: 200 Hz. Display Montage: Longitudinal Bipolar ('Double Banana’). For (C): Sensitivity 7 µV/mm, Low-Frequency Filter (LFF) 1.0 Hz, High-Frequency Filter (HFF) 70 Hz, Notch Filter 60 Hz ON, System: Nihon Kohden − Neurofax. Original Sampling Rate: 200 Hz. Display Montage: Longitudinal Bipolar ('Double Banana’).

A repeat MRI showed early acute, subacute, and chronic blood products in the right frontotemporal region beneath the craniotomy site. Consequently, the team decided to perform an Electrocorticography (ECoG)-guided resection of the right frontal convexity areas surrounding the previous hematoma cavity, which showed abundant polyspikes (Fig. 2). Postoperatively, EcoG confirmed termination of the polyspikes over the right frontocentral regions. Following surgery, the patient remained on continuous intravenous anesthetic sedation to maintain burst suppression. Post-operatively, over the course of 48 h, anesthetics were gradually weaned under continuous EEG monitoring. The status epilepticus was successfully terminated after surgical resection; however, the patient continued to experience frequent episodes of focal motor seizures with impaired awareness as well as electrographic seizures without clinical correlate and frequent epochs of periodic lateralized discharges (ictal-interictal continuum pattern). Given the persistence of focal status epilepticus despite resection, the decision was made to proceed with VNS implantation seven days after the surgical resection, which ultimately led to termination of the status epilepticus, however, the patient continued to have left upper extremity intermittent myoclonic jerks which had electrographic correlates in the form of polyspike discharges in the EEG as well as infrequent focal motor seizures with impaired awareness (Fig. 3c).

Upon follow up, the patient continued to report intermittent episodes of focal seizures with impaired awareness as well as EPC. The initial VNS settings post implantation were as follows − Normal mode Output Current: 2.0 mA, pulse width 500 µsec, Auto Stim current 2.0 mA and Magnet output was 2.25 mA. During the first follow-up after implantation, the VNS settings were adjusted as follows: Pulse width increased from 500 µsec to 750 µsec, Auto Stim current increased from 2.0 mA to 2.25 mA, and Magnet output was increased from 2.25 mA to 2.75 mA, after which no further adjustments were made to the VNS settings, however ASMs were adjusted on multiple follow up visits.

**Fig. 4:**
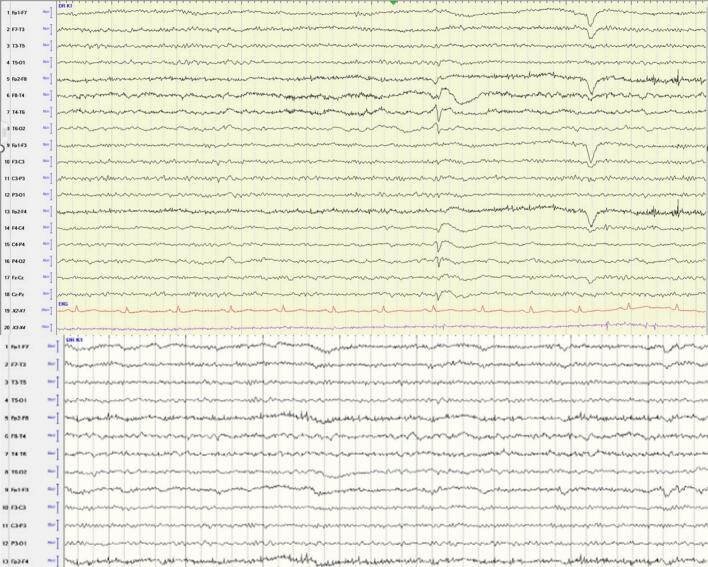
Video-EEG (vEEG) recordings demonstrating ictal correlate of myoclonic jerks and response to Botulinum toxin (BoNT) therapy. (A) vEEG epoch capturing a subtle left finger myoclonic jerk (video-confirmed) (B) vEEG epoch recorded after BoNT therapy, showing resolution of frequent epileptiform discharges. EEG Display Parameters (for A and B): Sensitivity 7 µV/mm, Low-Frequency Filter (LFF) 1.0 Hz, High-Frequency Filter (HFF) 70 Hz, Notch Filter 60 Hz ON, System: Nihon Kohden − Neurofax version QP-112AK Ver; Original Sampling Rate: 200 Hz. Display Montage: Longitudinal Bipolar ('Double Banana').

Post-VNS implantation and optimization, the patient experienced infrequent focal seizures with impaired awareness without progression to focal-to-bilateral tonic-clonic seizures. The dual approach of surgical resection and VNS implantation resulted in significant improvements in mental status and resumption of activities of daily living with support. However, she continued to have EPC involving the left upper extremity on the background of spastic hemiplegia. Subsequently, the patient underwent a repeat Epilepsy Monitoring Unit evaluation one year later, which captured persistent left UE myoclonic jerks preceded by runs of low amplitude polyspikes over the right frontocentral regions consistent with EPC (Fig. 4a).

For treatment of the left upper extremity spasticity and persistent EPC, she received three rounds of BoNT at approximately 3-month intervals over a period of one year, starting in March 2022. Adjustments to her anti-seizure medication (ASM) regimen occurred concurrently with the initiation of BoNT treatment in March 2022 and the subsequent follow-up visit (Table 1). Importantly, her ASM regimen remained stable, and no adjustments were made during the remainder of her BoNT treatment course (e.g. the second and third BoNT injections). This treatment resulted in a significant reduction of myoclonic jerks and spasticity, from occurring approximately every 5 min to only rare episodes, primarily during periods of stress or sleep onset. Ten months after the initial BoNT administration, a routine 3-hour EEG with additional surface electrodes on the left forearm captured only rare interictal epileptic discharges. Moreover, the surface electrodes placed over the left forearm did not capture any myoclonic jerks correlating with the interictal epileptic discharges (Fig. 4b). At the last follow-up, the patient reported occasional myoclonic jerks during stress and when she is falling asleep, no recurrence of focal seizures with impaired awareness. The patient's treatment history, including changes in anti-seizure medications (ASMs), vagus nerve stimulator (VNS) settings adjustments are summarized in Table 1**.**

**Table 1:** Anti-seizure Medication and VNS Adjustments.

**Date**	**VNS Changes**	**Medication Changes**
10/15/21	**Adjusted VNS settings:** Pulse width increased (500 → 750 µsec), Auto Stim current increased (2.0 → 2.25 mA), Magnet output increased (2.25 → 2.75 mA)	**Increased doses:** Keppra (**2000 mg BID**), Vimpat (**200 mg BID**), Phenobarbital (**64.8 mg 3 tabs qHS**), Phenytoin (**600 mg qHS**)
03/02/22	**VNS interrogated**; no changes made	**Slight increase in Phenytoin dose**, Referral to psychiatry to switch **Venlafaxine** to a different antidepressant
03/30/22	**VNS interrogated**, no changes reported	**Increased Phenytoin** (**100 mg 4 tabs qHS**), **Discontinued Topiramate**, Continued Phenobarbital (**64.8 mg qHS**) and Lacosamide (**150 mg BID**)
04/27/22	**VNS interrogated**; no changes made	**Increased Lacosamide** (**200 mg BID**), **Increased Keppra** (**2000 mg BID**)
05/11/22	No VNS changes	**Added Zonisamide:** 100 mg daily → increase to **200 mg** in 2 weeks → increase to **300 mg daily**
08/23/22	**VNS interrogated**; no changes made (Battery life: **25**–**50 %**)	**Discontinued Topiramate**, **Switched Phenytoin & Phenobarbital** from liquid to tablet form (same dose)
01/23/23	**VNS battery replaced (01/19/23)**, no changes made	No medication changes
02/01/23	**VNS interrogated**; no changes made	**24-hour EEG ordered** to assess myoclonic jerks before adjusting medications
06/14/23	No specific mention of VNS interrogation	**ASM levels checked** to assess for medication adjustments
10/31/23	No VNS check: patient hospitalized for lethargy	No medication changes
01/23/24	**VNS interrogated**; no changes made (Battery life: **25**–**50 %**)	**Changed Phenobarbital** to **60 mg 3 tabs qHS**, Plan to taper Keppra discussed but postponed

## 3 Discussion

Super-refractory status epilepticus can result from severe structural brain lesions, immunological disorders, gliotic scars, new onset super-refractory status epilepticus (NORSE), infectious diseases, drug or toxin exposures, and genetic disorders [5]. It is estimated that 10–20 % of all status epilepticus cases become SRSE [6]. Literature review revealed no case reports of patients undergoing the same sequence of interventions as our case, involving repeated brain surgeries, hematoma evacuation, EcoG resective surgery, and subsequent VNS implantation.

Surgical resection is recommended for prolonged SRSE with a definable radiological lesion or electrophysiological evidence of a focal onset, typically considered after two weeks of failed treatment [7,4]. Previous case studies have reported successful treatment of medically intractable status epilepticus with focal resection, subpial transection, or corpus callosotomy, with durations of status epilepticus before surgery ranging from 23 to 42 days [3]. Another study reported on five patients with refractory status epilepticus treated successfully with focal resection, showing full seizure control in four patients and significant reduction in seizure frequency in one patient [7].

VNS, an adjunctive treatment for drug-resistant epilepsy, has limited but promising efficacy in cessation of SRSE [8]. A systemic review of VNS in SRSE included 38 patients, with 74 % achieving cessation of RSE/SRSE with acute implantation [4]. Another review reported cessation in 76 % of generalized RSE patients and 25 % of focal RSE patients [8].

Botulinum neurotoxin (BoNT) therapy, a standard treatment for hyperkinetic movement disorders, inhibits neuromuscular transmission by preventing acetylcholine release at neuromuscular junctions [9]. BoNT has shown efficacy in reducing EPC, as evidenced by case reports of patients experiencing substantial reduction in myoclonus frequency and amplitude with ongoing BoNT treatment [10]. The case report showcases a series of 5 patients suffering from drug-resistant EPC with myoclonus of the face and arm were treated with repeated BoNT treatment over the course of almost 2 years. The results report a substantial reduction in frequency and amplitude of myoclonus in all patients as soon as the first application. They demonstrated a reduction of EPC by two-thirds, regardless of age, etiology of disease or duration. However, no EEG data were obtained after initiation of BoNT treatment so effects of BoNT therapy on EEG activity was not reported [9].

Our patient underwent a routine EEG approximately one year after starting BoNT treatment, revealing no electrographic seizures and only rare interictal discharges. Consistent with prior reports [9,10], our patient experienced reduced myoclonus following BoNT therapy. Importantly, our post-treatment EEG findings further support this benefit by demonstrating a corresponding reduction in epileptiform activity, offering electrophysiological evidence potentially underlying the clinical effect. While interpreting the specific contribution of BoNT is complex given the patient was on polytherapy and had undergone VNS implantation at that time, the results do offer some evidence. Although adjustments to the patient’s ASM regimen occurred around the time of the first BoNT injection (Table 1), potentially confounding the initial response to treatment, the subsequent improvement and sustained reduction in EPC frequency were observed following the second and third injections, during which her ASM’s were not adjusted. This observation lends support to an independent therapeutic contribution from BoNT in managing the patient’s EPC. Furthermore, the routine EEG performed approximately ten months after initiating BoNT revealed no EEG seizures and only rare interictal discharges, correlating with the sustained clinical improvement. Clinical observation demonstrated that BoNT reduced EPC, the exact mechanism is unclear, although BoNT could play a role in modulating the epileptic networks resulting in a decrease in EPC [11].

## 4 Conclusion

The management of SRSE, particularly prolonged SRSE, is challenging. In severe cases, when conventional medical treatments fail, sequential surgical resection followed by VNS implantation shows promise, despite limited reporting in case studies. Managing residual EPC with BoNT may be a possible avenue, however, the results obtained in this case report are only observational.

## Ethical statement

This case report was conducted in accordance with ethical guidelines. Written informed consent was obtained from the patient directly for the publication of this report and associated images. No identifying details have been disclosed to maintain the patient’s confidentiality. Institutional Review Board (IRB) approval was obtained from Kern Medical IRB and a copy is available upon request if necessary.

## CRediT authorship contribution statement

**N. Zalmay:** Writing – review & editing, Writing – original draft, Methodology, Formal analysis, Data curation, Conceptualization. **G. Nune:** Data curation. **C.N. Heck:** Resources. **K.T. Dao:** Writing – review & editing. **B.T. Ly:** Writing – original draft, Conceptualization. **J. Ipe:** Writing – original draft. **C.Y. Liu:** Conceptualization. **H.P. Kunhi Veedu:** Supervision, Investigation, Formal analysis, Conceptualization.

## Declaration of competing interest

The authors declare that they have no known competing financial interests or personal relationships that could have appeared to influence the work reported in this paper.
